# Metagenomic analysis of medicinal *Cannabis* samples; pathogenic bacteria, toxigenic fungi, and beneficial microbes grow in culture-based yeast and mold tests

**DOI:** 10.12688/f1000research.9662.1

**Published:** 2016-10-07

**Authors:** Kevin McKernan, Jessica Spangler, Yvonne Helbert, Ryan C. Lynch, Adrian Devitt-Lee, Lei Zhang, Wendell Orphe, Jason Warner, Theodore Foss, Christopher J. Hudalla, Matthew Silva, Douglas R. Smith

**Affiliations:** 1 Medicinal Genomics Corporation, Woburn, MA, 01801, USA; 2 ProVerde Laboratories, Milford, MA, 01757, USA

**Keywords:** Cannabis, safety, PathogINDICAtor-qPCR, 3M-Petrifilm, Biomérieux-TEMPO, Illumina, metagenomics, microbiome

## Abstract

**Background**: The presence of bacteria and fungi in medicinal or recreational *Cannabis *poses a potential threat to consumers if those microbes include pathogenic or toxigenic species. This study evaluated two widely used culture-based platforms for total yeast and mold (TYM) testing marketed by 3M Corporation and Biomérieux, in comparison with a quantitative PCR (qPCR) approach marketed by Medicinal Genomics Corporation.

**Methods**: A set of 15 medicinal *Cannabis* samples were analyzed using 3M and Biomérieux culture-based platforms and by qPCR to quantify microbial DNA. All samples were then subjected to next-generation sequencing and metagenomics analysis to enumerate the bacteria and fungi present before and after growth on culture-based media.

**Results**: Several pathogenic or toxigenic bacterial and fungal species were identified in proportions of >5% of classified reads on the samples, including *Acinetobacter baumannii, Escherichia coli, Pseudomonas aeruginosa, Ralstonia pickettii, Salmonella enterica, Stenotrophomonas maltophilia, Aspergillus ostianus, Aspergillus sydowii, Penicillium citrinum *and *Penicillium steckii.* Samples subjected to culture showed substantial shifts in the number and diversity of species present, including the failure of *Aspergillus* species to grow well on either platform. Substantial growth of *Clostridium botulinum* and other bacteria were frequently observed on one or both of the culture-based TYM platforms. The presence of plant growth promoting (beneficial) fungal species further influenced the differential growth of species in the microbiome of each sample.

**Conclusions**: These findings have important implications for the *Cannabis* and food safety testing industries.

## Introduction

Plant associated microbes may present risks of infectious illness for human end consumers. However, many plant-associated microbes may provide benefits for plant cultivation in terms of growth stimulation, insect or microbial resistance, or may simply be neutral passengers ^1– 3^. The microbiome of *Cannabis* leaves and flowers includes bacteria and fungi residing on the exterior surface of these tissues (epiphytes) as well as those residing within the plant tissues (endophytes). While epiphytic microbes may originate from many sources like aerosols, dusts and liquids, or via human contact, endophytes typically gain entry from the rhizosphere via root junctions, and subsequent translocation through the xylem ^4, 5^. Considering this and the known impact that the soil and root microbiome has on plant growth and development ^6, 7^, all sources of microbial inputs, including below ground compartments should be considered important for optimal *Cannabis* growth and consumer safety ^8^.

Studies on the natural Cannabis microbiome have identified several species of culturable endophytic fungi, including *Penicillium citrinum, Penicillium copticola* (a member of the citrinum section ^9^) and several *Aspergillus* species ^10, 11^. Similar studies looking at culturable bacterial endophytes identified nearly a dozen isolates from the Bacillus clade and two mycobacteria ^1^. Of those *Bacillus* species, *B. subtilis*, *B. lichenoformis* and *B. pumilis* have been isolated as endophytes and have been shown to be beneficial to growth in other plant species ^12– 14^. Finally, a recent investigation of the fungal microbiome in a number of dispensary-derived *Cannabis* samples identified numerous species including some toxigenic Penicillia and Aspergilli ^15^. While there have not been any reported cases of *Cannabis*-related mycotoxin poisoning resulting from *Penicillium* infections, there have been numerous reported cases of serious or fatal pulmonary Aspergillosis associated with marijuana smoking in immunocompromised patients ^16– 18^. A multistate outbreak of Salmonellosis has also been reported ^19, 20^. Denver’s Department of Environmental Health has also issued warnings related to *Cannabis* extracts and *Clostridium botulinum* ^21^.

State *Cannabis* markets rely on a patchwork of testing regulations to protect patients and consumers. In terms of microbial testing, these vary widely from state to state. States such as Maine, Michigan, and Arizona currently do not impose testing regulations, while several states such as Connecticut, Massachusetts and New Mexico have adopted regulations based on the United States Pharmacopeia (USP) and American Herbal Pharmacopeia (AHP) recommended guidelines ^22^. Specifically, the AHP recommends appropriate methods for testing microbial loads be adopted from the FDA Bacteriological Analytical Manual ( http://www.fda.gov/Food/FoodScienceResearch/LaboratoryMethods/ucm2006949.htm). State regulators frequently use AHP guidelines to set limits of 10 ^5^ CFU/g for Total Aerobic Bacteria (TAC), 10 ^4^ CFU/g for Total Yeast and Mold (TYM), 10 ^3^ CFU/g for Total Coliform and *Enterobacteriaceae* and < 1 CFU/g for pathogenic *E*. *coli* and *Salmonella* species. The AHP states, “It is important to note that microbial and fungal values do not typically represent pass or fail criteria and recommended limits may require adjustment over time.” New York and Hawaii specify some additional genera for testing such as *Aspergillus, Klebsiella, Pseudomonas, Streptococcus, Mucor,* and *Penicillium*. A few States require that testing laboratories follow the procedures outlined in the USP for microbiological examination of non-sterile products. Others allow testing laboratories to choose from a wide variety of technologies designed for the food testing industry. However, there is no peer-reviewed research supporting the effectiveness and validity of any of these protocols for *Cannabis* microbial testing. Furthermore, no studies to date have examined the impact of beneficial endophytes on the *Cannabis* microbiome and on microbial testing results.

Here we present a next generation sequencing survey of DNA sampled directly from cured cannabis flowers before and after culturing using 3M Rapid Yeast and Mold Petrifilm ^TM^, the Biomérieux Tempo ^®^ Total Yeast and Mold platform, and qPCR analysis using Medicinal Genomics ITS2-based TYM and 16S-based TAC assays. Sequencing and analysis of the fungal ribosomal operon internal transcribed spacer ^23, 24^ (ITS2) and the bacterial 16S ribosomal RNA gene V3 and V4 hypervariable regions ^25^ (16S) allowed us to identify bacterial and fungal genera and species present in each case. The results highlight some organisms of concern and demonstrate that major fungal and bacterial compositional changes occur during culture-based TYM testing. 

## Methods

### Samples, culture-based assays and DNA purification

Cannabis samples were derived from seven recently-established indoor growth facilities in Massachusetts, Maine and Rhode Island. Samples were prepared and placed into culture on 3M Petrifilm ^TM^ Rapid Yeast and Mold Count Plates (40–72 h) and Biomérieux TEMPO ^®^ YM cards (70–76 h) at 25 ± 1.0°C, according to the manufacturer’s instructions. All samples but two were also analyzed using Biomérieux TEMPO ^®^ AC cards to enumerate aerobic bacterial counts. For qPCR, *Cannabis* samples (250 ± 30 mg) were placed in Whirl-Pak ^®^ bags and massaged in 3.55 ml Trypticase Soy Broth (TSB; American Bioanalytical) for 1 minute. DNA was then extracted using SenSATIVAx reagents (Medicinal Genomics part #420001), as described previously ^15^ and eluted with 50 μL ddH20. DNA was similarly extracted after growth on the two culture based platforms as described above. Colonies grown on 3M plates were scraped off into 285 μL of ddH2O, and 190 μL of those samples, or samples grown in TEMPO cartridges (liquid culture), were extracted using SenSATIVAx as above. Fungal species stocks from the American Type Culture Collection (ATCC) were reconstituted and incubated at the appropriate temperature, as recommended by ATCC product documentation. Cultures of ATCC strains were then grown in 5ml TSB for 5 days at room temperature and checked visually for turbidity. Serial dilutions were plated on 3M PetrifilmTM Rapid Yeast and Mold Count Plates, incubated at room temperature, and counted after 3–5 days. Colonies were scraped off the plates and DNA was then extracted as described above.

The cannabis samples used for this study were collected within the regulatory framework for the individual State Medical Marijuana programs by ProVerde Laboratories; an accredited ISO/IEC 17025:2005 cannabis safety testing laboratory. The purified DNA, which is not a schedule I substance, was tested to verify that the hydrophilic DNA purification does not contain hydrophobic cannabinoids and is therefore in accordance with the Hemp Associates vs DEA regarding hemp fiber shipment within the United States. Since all activities that involved handling of material containing cannabinoids was within the individual state requirements, no federal (FDA or DEA) registration or permission was required.

### Total yeast and mold and total aerobic bacteria qPCR assays

DNA samples extracted directly from *Cannabis* samples, or after growth on the two culture-based platforms, were subjected to qPCR analysis. Quantitative PCR was performed using a commercially available TYM assay (TYM-PathogINDICAtor, Medicinal Genomics, Woburn MA), or TAC assay (TAC-PathogINDICAtor, Medicinal Genomics, Woburn, MA) in a Bio-Rad CFX 96 Touch qPCR instrument, according to the manufacturer’s instructions.

### Primers used for PCR and sequencing

PCR was performed using 5μL of DNA (3ng/μL) 12.5μL 2X LongAmp (NEB) with 1.25 μL of each 10 μM MGC-ITS3F and MGC-ITS3R primer or MGC-TAC_F and MGC-TAC_R primer (MGC-ITS3F: TACACGACGTTGTAAAACGACGCATCGATGAAGAACGCAGC), (MGC-ITS3R: AGGATAACAATTTCACACAGGATTTGAGCTCTTGCCGCTTCA), (MGC-TAC_F: TACACGACGTTGTAAAACGATCCTACGGGAGGCAGCAGT) and (MGC-TAC_R: AGGATAACAATTTCACACAGGGGACTACCAGGGTATCTAATCCTGTT) with 10μL ddH20 for a 25 μL total reaction. An initial 95°C 5-minute denaturation step was performed followed by 25 cycles of 95°C for 15s and 65°C for 90s. Samples were purified with 75 μL SenSATIVAx, washed twice with 100 μL 70% EtOH and bench dried for 5 minutes at room temperature. Samples were eluted in 25 μL ddH20.

### Library preparation using Nextera

The 16S amplicon targeted by the MGC primers (spanning the V3 and V4 hypervariable regions) is approximately 460 bp in size, and ITS2 amplicons from different fungal species are known to vary in size from ~0.5–1 kilobases. To enable representative coverage across the entire amplicon for sequencing and analysis of each sample, we enzymatically fragmented the amplicons to ~300 bp average size. Fragmentation was accomplished and DNA libraries were constructed using the commercially available Nextera Library Prep Kit (Illumina). 6ng of purified PCR product, 5 μL of TD buffer, 0.1 μL of TD enzyme and 3.9 μL ddH20 was combined for a total of 10 μL. The reaction was incubated at 55°C for 30 minutes followed by a 10°C hold. The reaction plate was immediately removed from the thermal cycler and purified with 15 μL of Agencourt Ampure XP (Beckman Coulter), washed twice with 200 μL 70% EtOH and bench dried for 10 minutes at room temperature. Samples were eluted in 25μL 10mM Tris-HCl.

### Library PCR and Illumina sequencing

17.5 μL of 2X Q5 polymerase (NEB) was added to 10μL of purified DNA with 2.5 μL of i7 Nextera index primer, 2.5 μL L of i5 Nextera index primer, 0.5 μL of ILMN1 primer (50 μM), 0.5 μL of ILMN2 primer (50 μM), 1 μL 5-methyl-dCTP (10 μM) and 0.5 μL H _2_O. After an initial 72°C for 3 minutes and 98°C for 30 s, the library was amplified for 12 cycles of 98°C for 10 s, 63°C for 30 s, 72°C for 1 minute and a 10°C hold. Use of methylated nucleotides for PCR decontamination is described previously ^26, 27^. PCR samples were purified by mixing 52.5 μL of Agencourt Ampure XP into the PCR reaction. The samples were placed on a magnet for 15 minutes until the beads cleared and the supernatant could be removed. Beads were washed twice with 200 μL of 70% EtOH. Beads were left for 10 minute to air dry and then eluted in 25 μL of 10 mM Tris-HCl. 5 μL of each PCR product was pooled and quantified with a Qubit (Thermo) for proper dilution onto MiSeq version 2 chemistry according to the manufacturers’ instructions. 2×150 bp reads were selected to obtain maximal ITS2 sequence information. 

### Analysis

2×150 bp reads were de-multiplexed with Illumina software bcl2fastq v2.17.1.14. Sequences were classified at the Family, Genus and Species level by discriminative k-mer analysis using CLARK-S ^28^ with the NCBI/RefSeq bacterial database and taxonomy, or UNITE ^29^ fungal database and taxonomy. *Cannabis* chloroplast and mitochondrial sequences were included in the bacterial and fungal databases since they amplify with the 16S rRNA primers used, and the Nextera fragmentation process used in our lib prep may incorporate high copy number sequences even without amplification. *Cannabis* mitochondrial sequences generally comprised a large fraction of the classified reads (up to 97%) in DNA derived from plant material. The Cannabis reads were subtracted out to enable enumeration of the bacterial species down to 1% of classified non- *Cannabis* reads.

Sequences were alternatively classified by BLAST analysis of operational taxonomic units (OTUs) generated by clustering at the ≥ 97% sequence similarity level using USEARCH8 ^30^. Each set of paired-end reads were merged using fastq_merge pairs ^31^. We used cutadapt to trim primer and adaptor regions from both ends ( http://cutadapt.readthedocs.io/en/stable/guide.html). Sequences were quality trimmed to have a maximum expected number of errors per 100 bases of less than 0.1 (Q30). OTUs with membership of at least 200 sequences were included in downstream analyses, and BLAST hits with less than 97% query coverage and 97% identity were discarded. Analyses of the USEARCH OTUs were performed in R ( https://www.r-project.org). Each library was normalized by the total number of OTUs found. OTUs were associated with microbes based on the name and description provided by NCBI. R ^2^ values were calculated by adjusted linear regression in R or by embedded formulas in Excel. In order to mitigate the large effect of noise in samples with low OTU counts, specificity analysis was done after pooling the un-normalized data.

## Results

### Quantitative PCR and colony counts before and after culture

Summary results from the different testing platforms evaluated in this study for 15 samples with complete data are presented in Table 1. The samples were evaluated with Medicinal Genomics’ PathogINDICAtor ITS2-based TYM-qPCR and 16S-based TAC-qPCR assays directly from extracted plant material (Before), and from recovered medium after culture on the Biomérieux Tempo instrument using YM sample cards (After BMX). Samples were also evaluated directly using the Biomerieux instrument with Tempo YM and AC cards, or on 3M Rapid Total Yeast and Mold Count Plates (3M TYM). Results in bold type and shaded boxes indicate failed tests following the limits set for Massachusetts medicinal *Cannabis*.

**Table 1.** Quantitative PCR and colony count results. Column 1: sample number; Column 2: results TYM-qPCR signals in terms of quantification cycle (Cq); Column 3: colony counts for 3M TYM plates; Column 4, inferred colony counts from BMX YM cards, Column 5: TYM-qPCR Cq signals after culture in the BMX YM system; Column 6: TAC-qPCR Cq signals from extracted plant material; Column 7: inferred colony counts from BMX AC cards, and Column 8: TAC-qPCR Cq signals after culture in BMX YM cards. Results in bold type and shaded boxes indicate failed tests. Abbreviations: BMX: Biomerieux, TYM: total yeast and mold, YM: yeast and mold, TNTC: too many to count, TAC: total aerobic count, AC: aerobic count, n.d.: not done. The AC and TYM failure thresholds for colony counts on the 3M and Biomerieux platforms are 100,000 CFU/g and 10,000 CFU/g respectively. The TAC and TYM qPCR failure thresholds are Cq ≤ 21 and Cq ≤ 26, respectively.

Sample	TYM- qPCR Cq Before	3M TYM CFU	BMX YM CFU	TYM-qPCR Cq After BMX	TAC-qPCR Cq Before	BMX AC CFU	TAC-qPCR Cq After BMX
1	40+	0	2.8,000	40+	26.34	**>490,000**	**20.36**
2	40+	0	590	40+	25.12	34,000	**15.2**
3	**24.52**	**TNTC**	**490,000**	**19.76**	24.22	48,000	**16.19**
4	27.7	**80,000**	**110,000**	**20.73**	**21.09**	**>490,000**	**16.01**
5	**23.25**	**TNTC**	**490,000**	**22.24**	24.22	78,000	**16.19**
6	**20.48**	**240,000**	**250,000**	**20.73**	40+	<100	30.38
7	40+	0	<100	40+	29.4	310	27.54
8	40+	0	100	26.79	24.05	<100	26.93
9	40+	1,000	440	**25.45**	40+	450	26.65
11	40+	10,000	**12,000**	**17.77**	27.38	n.d.	27.8
12	40+	**14,000**	**14,000**	**19.54**	30.55	n.d.	28.56
13	40+	**28,000**	**19,000**	**21.21**	35.33	<100	30.39
14	40+	**TNTC**	**250,000**	**20.49**	26	210	29.97
15	40+	**211,000**	**140,000**	**20.08**	23.98	100	30.12
16	40+	**TNTC**	**210,000**	**22.3**	25.28	100	31.63
	*Fail: ≤ 26*	*Fail: > 10,000*	*Fail: > 10,000*	*Fail: ≤ 26*	*Fail: ≤ 21*	*Fail: > 100,000*	*Fail: ≤ 21*

Overall, the BMX TYM platform failed the highest number at 67% (10/15); the 3M TYM platform failed 60% (9/15), and the qPCR TYM failed 20% (3/15). The failure rates for the BMX AC and qPCR TAC assays were 13% (2/15) and 7% (1/15), respectively. An additional set of TYM qPCR tests were performed after growth on the BMX platform, resulting in 12/15 failures and confirming the presence of live, culturable fungi in 80% of the samples. The 3M TYM and BMX YM systems performed similarly in terms of pass/fail, with only one discrepancy, which had a value close to the failure threshold. The TYM-qPCR assay passed seven samples that failed on at least one of the two culture-based platforms. One of those (sample 4) had an elevated quantitation cycle (Cq) value approaching the failure threshold; the rest (samples 11–16) gave high Cq values, indicating very low fungal DNA levels ( Table 1).

### Metagenomic sequencing and analysis results

The sequencing data generated for this project are available at the NCBI short read archive; see Dataset 1 (Table I) for accession numbers and URLs. A summary of the CLARK-S classification results for each of the 15 samples, directly from plant material (before), or after culture on the 3M or BMX platforms, is provided in Dataset 1 (Table II: CLARK-S output for bacterial species analysis with read counts, Table III: matrix file with % classified reads at the species level for all TAC samples, Table IV: matrix file with % classified reads down to 1% at the species level from selected TAC samples used to generate charts, Table V: CLARK-S output for TYM analysis with read counts, Table VI: matrix file with % classified reads for all TYM samples, Table VII: matrix file with % classified reads down to 1% from selected TYM samples used to generate charts, Table VIII: matrix file with % classified reads down to 1% at the genus level from the same selected TAC samples as in Table IV).

While the sequencing assay provides approximate intra-sample quantitation, it does not support inter-sample quantitation ^32^. The sequencing procedure utilizes two PCR steps instead of the single PCR step used in qPCR (and does not utilize an internal probe for signal generation). Sample quantities are normalized prior to the Nextera reaction to ensure consistent shearing. These procedures are optimized to yield 1 million reads or more per sample for high sensitivity, but the read numbers are not proportional to microbial counts in the starting samples. Instead, the classified read counts and percentages simply indicate the genera or species present at detectable levels and their approximate proportions (with the caveat that the target amplicons from some species may amplify with lower efficiency owing to primer mismatches or extremes of G+C content). The qPCR Cq measured directly from extracted plant material provides the best inter-sample comparative metric. BLAST results from clustered OTUs were used to confirm prevalent species assignments on a case-by-case basis, but the results are not presented here owing to the very large number of OTUs generated by the USEARCH software (>12,000 across the full sample set).

Sequencing reproducibility: 14 frozen samples were amplified with ITS2 primers and sequenced 30–60 days apart; 13 of the comparative R square values for classified fungal species were greater than 0.999 and the remaining one was 0.966. Similarly, 20 frozen samples were amplified with 16S primers and sequenced 30–60 days apart; 18 of the comparative R square values for classified bacterial species were greater than 0.999 and the remaining two averaged was 0.998. These data imply highly reproducible genomic surveys of the amplified DNA present. No Template Controls (NTC) were also tested, producing very high Cq readings (>35) and very few classified reads (251 with TAC primers and 61 with TYM primers) controlling for the possibility of labware contamination contributing to the observed signals.

Specificity: To verify the specificity of the analysis for accurate discrimination between bacterial and fungal genera, we ran CLARK-S against the bacterial and fungal databases separately at the genus level using either 16S or ITS2 reads. There were 13,913,520 16S reads classified as bacterial, 2,293 16S reads classified as fungal, 6,220,745 ITS2 reads classified as fungal, and 241,351 ITS2 reads classified as bacterial ( Dataset 1, Tables V and IX–XI; Table IX: genus level CLARK-S read counts for 16S reads against the fungal database, Table X: genus level CLARK-S read counts for ITS2 reads against the bacterial database, Table XI: genus level CLARK-S read counts for 16S reads against the bacterial database). From this we calculate the specificity *(true neg/(false pos + true neg)* of 16S analysis as 0.963 *[=ITS2 reads classified as fungal/(ITS2 reads classified as fungal+ITS2 reads classified as bacterial)]* and that of the ITS2 analysis as 0.9997 *[=16S reads classified as bacterial/(16S reads classified as bacterial+16S reads classified as fungal)]*.

Pairs of samples from three of the seven growers were highly similar in their combined bacterial and fungal species prevalence as indicated by high correlation coefficients (CC): CC=0.92 for samples 1 and 2, CC=0.94 for samples 11 and 12, and CC=0.97 for samples 6 and 14. There was also moderate correlation between samples 6, 14 and 9, a third sample from the same grower: CC=0.66 for samples 6 and 9, CC=0.64 for samples 9 and 14. These samples represent different strains from the same grow and likely share similar soil environments.

### Bacterial growth on culture-based TYM platforms

Six samples (numbers 11–16) failed in the BMX TYM test, but passed the MGC qPCR TYM test with low signals (Cq >40). Five of those (numbers 11, 12, 14–16) had elevated qPCR TAC signals, suggesting that the growth of bacteria could be contributing to colony counts and failures in the culture-based TYM tests. Sequencing results for each of those samples, before and after culture in BMX medium, confirm the presence of actively growing bacteria, and reveals the bacterial genera that are primarily responsible for the TAC-qPCR signals: *Bacillus* and *Clostridium* in sample 11 (~73% of classified reads, collectively), and *Bacillus*, *Clostridium* and *Ralstonia* in samples 14–16 (78–83% of classified reads, collectively in the each of the three samples). A different set of genera were observed after culture on 3M media: *Ralstonia* and *Leifsonia* in sample 11 (86% of classified reads, collectively), and *Xanthomonas, Ralstonia* and Streptococcus in samples 14–16 (61–75%, collectively in each sample).

**Figure 1.**
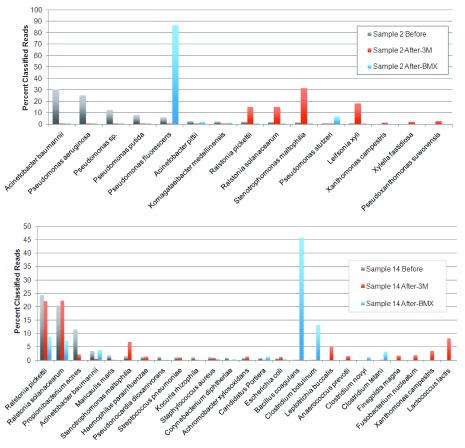
Genomic profiles of before and after culturing. Comparison of classified read percentages for bacterial 16S DNA on samples 2 and 14, before and after culturing on 3M and BMX media. The results represent all species observed down to 1% of classified reads. Large shifts in species prevalence are seen after growth on the two culture-based platforms.

All of the samples underwent a change in species composition after growth on the BMX or 3M yeast and mold platforms. Three of the 15 samples (numbers 5, 15 and 16) produced a similar distribution of species on the BMX and 3M platforms, with correlation coefficients (CC) of 0.41–0.82. The results from the remaining 80% of the samples, however, were strikingly different on the two platforms (CC: -0.03-0.21). Representative results from two of those samples, numbers 2 and 14, are shown in Figure 1.

Significant levels of *Bacillus coagulans* and *Clostridium botulinum* (a toxigenic pathogen) were observed together in two thirds of the samples (numbers 6–9 and 11–16) after incubation in the hermetically sealed cards of the BMX TYM platform. These organisms were detected before growth at very low levels (0.5% or less), indicating the presence of viable cells or spores in the samples. They were not detected at significant levels after growth on the 3M platform.

Other potentially pathogenic bacterial species that were detected at proportions of >1% of classified bacterial reads on plant material before growth include: *Acinetobacter baumannii, Acinetobacter pitti, Corynebacterium diphtheriae, Coxiella burnetii, Escherichia coli, Propionibacterium acnes, Pseudomonas aeruginosa, Ralstonia pickettii, Salmonella enterica, Staphylococcus aureus, Stenotrophomonas maltophilia, and Streptococcus pneumoniae*. Some of these species, and others, were observed to grow differentially on the BMX and 3M platforms. Species that grew well on 3M but not BMX included *S. maltophilia* and *Leifsonia xyli*; those that grew well on BMX but not 3M included *C. botulinum, B. coagulans, Pseudomonas fluorescens* and *C. tetani.* Factors that may contribute to this are the presence of chloramphenicol (Cm) and possible low oxygen levels in the BMX platform. *S. maltophilia* is Cm sensitive and *P. fluorescens* is Cm resistant. *C. botulinum* and *C. tetani* are obligate anaerobes and *B. coagulans* is a facultative anaerobe.

### Differential growth of toxigenic and beneficial fungi

The concordance between the two culture based platforms was much higher overall for fungi than for bacteria. The distribution of fungal species observed after growth on the BMX and 3M platforms was highly similar for nine of the 15 samples (cc 0.98-1.0), and low to moderate for another three samples (cc: -0.02-0.49). The remaining three samples did not include any fungi that could be classified at the species level. The following toxigenic fungi were detected levels at >1% of classified reads in at least one sample: *Aspergillus fumigatus, Aspergillus ostianus, Aspergillus sydowii, Penicillium citrinum, Penicillium commune, and Penicillium steckii.*

**Figure 2.**
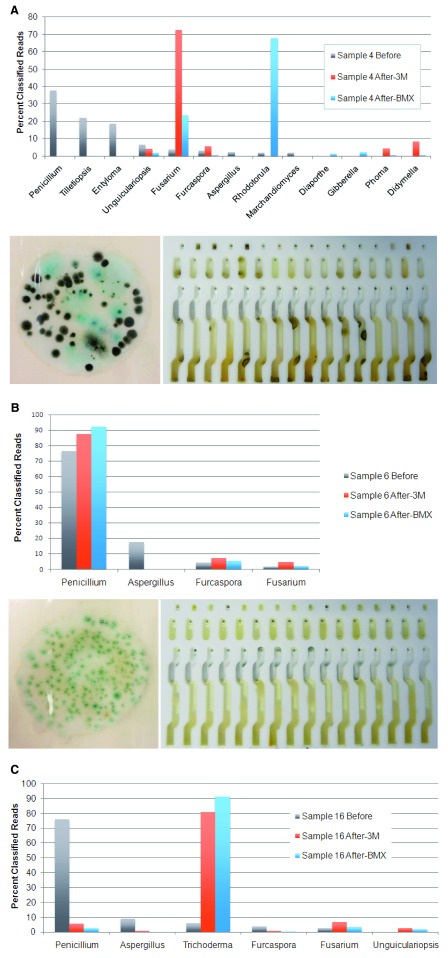
**A) TYM platform discordance before and after growth.** Results from sample 4 showing the percentage of reads classified into fungal genera based on sequencing of TYM ITS2 amplicons directly from the plant (Before), or after growth on the 3M or BMX platforms. The lower part of the figure shows the colonies observed on 3M media (left) and appearance of the BMX YM card (right) after growth. **B) Poor growth of *Aspergillus* species.** In 12/15 cases where *Aspergillus* species are detected by ITS2 sequencing, they do not grow on 3M or BMX media (results from sample 6). The lower part of the figure shows the colonies observed on 3M media (left) and appearance of the BMX YM card (right) after growth. **C) Trichoderma antagonism.** Penicillium species are present in material extracted directly from the plant in sample 16, but are displaced by Trichoderma after growth on 3M or BMX media.

We expected that all fungal species would grow effectively on the 3M and BMX TYM platforms, but there were some notable exceptions. First, we observed that although *Aspergillus* species were present in 15 plant samples (average proportion: 25% of classified reads), they were only detected at low levels in three samples after culturing on either 3M or BMX media (average proportion: 1.1% or 0.4% of classified reads, respectively). Representative results from two such samples are shown in Figure 2. Second, *Penicillium* was the most prevalent genus observed before and after growth on both platforms, with the most prevalent species classifications being *P. citrinum* and *P. olsonii*. However, although *Penicillium* species were present at significant levels in sample 16 (76% of classified reads; Figure 2C), they did not grow well on either platform in this sample (2.7–5.6% of classified reads). Instead, substantial growth of *Trichoderma* species, primarily *T. hamatum*, was observed (80–90% of classified reads). *T. hamatum* is one of several *Trichoderma* species that have been shown to inhibit the growth of *Penicillium* and other toxigenic fungi ^33, 34^. Apparent competitive growth inhibition of *Penicillium* species was also observed in sample 4 where there was substantial growth of *Fusarium* species (23–72% of classified reads; Figure 2A), and in samples 1, 2 and 7 where there was substantial growth of *Saccharomyces* species (57–82% of classified reads).

While the qPCR and sequencing assays are capable of detecting free DNA, all of the samples tested in this study appear to contain live spores or microbes. Even in the one sample (number 6) where the TYM-qPCR Cq did not decrease after growth in BMX media, the proportions of fungal species changed and TAC qPCR demonstrated growing bacteria with a 10 Cq decrease (from over 40 to 30.4) after culture.

### Comparative growth of *Aspergillus species* and other fungi on 3M media

**Figure 3.**
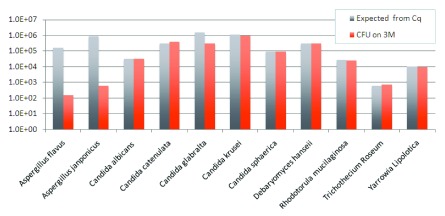
The following 11 species were grown at RT. *Candida catenulata*: ATCC 10565, *Candida sphaerica*: ATCC 8565, *Candida krusei*: ATCC 28870, *Candida albicans*: ATCC 10231, *Candida glabralta*: ATCC 15545, *Yarrowia lipolytica*: ATCC 18944, *Rhodotorula mucilaginosa*: ATCC 4557, *Debaryomyces hanseii*: ATCC 10623, Trichothecium Roseum: ATCC 90473, Aspergillus *japonicus*: ATCC 16873, *Aspergillus flavus*: ATCC 16870. Aspergillus demonstrates log scales lower growth at RT than most other yeast. “Expected” is the inferred CFU count from the Cq measurement using the formula CFU/g = 10 ^[(42.185 – Cq Value)/3.6916]^.

To further evaluate the ability of Aspergillus species to grow on 3M Rapid TYM Petri-Films, we plated 10 fungal monocultures from ATCC stocks and measured the concordance between qPCR Cq and 3M CFU ( Figure 3). The *Aspergillus* species CFU counts are approximately three orders of magnitude lower than expected based on Cq estimates that were developed and optimized by plating cultured cells of other species. Excluding the two *Aspergillus* species, the correlation between CFU/g and Cq is 0.71. The one other outlier in these data is *Candida glabralta.* The correlation between CFU per gram of plant material and Cq is 0.99 across the remaining eight different fungal species.

Raw data of metagenomic analysis of medicinal Cannabis samples All data files supporting this work are provided. Click here for additional data file. Copyright: © 2016 McKernan K et al. 2016 Data associated with the article are available under the terms of the Creative Commons Zero "No rights reserved" data waiver (CC0 1.0 Public domain dedication).

## Discussion

The samples selected for this study were derived from seven newly established indoor *Cannabis* growth facilities located in a humid coastal environment (Eastern Massachusetts, Maine and Rhode Island). They were enriched for samples that failed on either or both the 3M and BMX platforms, which are commonly used to test for bacteria, yeast and mold in the industry. Quantitative PCR was evaluated as a third approach to hopefully resolve discrepancies. The high failure rate observed in this study should not be taken as representative of industry-wide averages, which have been reported elsewhere ^15, 35^. The sample set provided an opportunity to investigate the diversity of species that grow in different culture-based platforms as well as to characterize the microorganisms that were responsible for the sample failures.

Metagenomic sequencing data were collected on 15 samples, directly from plant material and after culture on both the 3M and BMX platforms. The sequencing results demonstrate substantial shifts in presence and abundance of bacterial and fungal species after growth on the two platforms. Thus both of the culture-based platforms are detecting and enumerating only a subset of the species present, and the final composition of microbes after growth is markedly different from the starting sample. Most concerning is the frequent identification of bacterial species in systems designed for the exclusive quantification of yeast and mold, as quantified by elevated TAC Cq values after culture in the BMX TYM medium. These observations call into question the specificity claims of these culture-based testing platforms. The presence of bacterial colonies on TYM growth plates or cards may falsely increase the rejection rate of *Cannabis* samples for fungal contamination, and induce growers to increase the use of fungicides unnecessarily.

Classified reads corresponding to many pathogenic and/or toxigenic bacteria and fungi were detected on plant material, including the following at proportions of over 5%: *Acinetobacter baumannii, Acinetobacter pittii, Escherichia coli, Propionibacterium acnes, Pseudomonas aeruginosa, Ralstonia pickettii, Salmonella enterica, Stenotrophomonas maltophilia, Penicillium citrinum, Aspergillus ostianus, Penicillium steckii and Aspergillus sydowii.* While the proportions of classified reads corresponding to these organisms were generally low, there were several striking exceptions: >10–35% *R. picketti* in 9/15 samples, 97% *S. maltophilia* in one sample, 41% *E. coli* in one sample, 16–35% *A. baumanii* in two samples, 10–85% *P. olsonii* in five samples, 10–72% *P. citrinum* in 13 samples, and 21% *A. ostianus* in one sample. The CLARK-S classification software has been reported to have very high sensitivity and precision for sequence assignments ^28, 36, 37^. Nevertheless, further work is required to confirm these species assignments and to check for the presence of toxins that may be produced by these microbes. The observations certainly call into question the wisdom of species-agnostic microbial quantitation for a product like medicinal *Cannabis,* which is used by many seriously ill or immunocompromised patients.

Cross-platform comparisons demonstrate that certain bacteria and fungi grow well on 3M plates, but not on BMX, or *vice versa*. There are certainly differences in terms of the media. For example, BMX medium includes chloramphenicol to suppress bacterial growth, and uses sealed growth chambers that may limit oxygen availability. The observation of anaerobic *Clostridium* species such as *C. botulinum* in proportions up to 35% of bacterial reads at the genus level on the BMX platform along with *B. coagulans,* a facultative anaerobe, suggests that the sealed BMX YM cards generate anaerobic conditions. *B. coagulans* is rhizobacterium that has been reported to promote growth in *Solanum* seedlings in concert with mycorrhizal fungi ^38^.

*Clostridium botulinum* was only detected at very low levels before growth on BMX medium, and was not detected on 3M plates. Previous white papers have suggested *C. botulinum* is not a threat in *Cannabis* due to its anaerobic nature ( http://cannabissafetyinstitute.org/wp-content/uploads/2015/06/Microbiological-Safety-Testing-of-Cannabis.pdf). However, *C. botulinum* should not be considered an irrelevant threat in *Cannabis* because it is known to vascularize as an endophyte in plants and produce pasteurization resistant spores ^39^. Additionally, proximity between cultivation and processing may lead to contamination of finished products such as emulsified oils or concentrated extracts containing water. Media such as these provide anaerobic conditions and nutrients sufficient for *C. botulinum* and other anaerobes to thrive. This is most threatening to indoor cultivation facilities which also process, store, and package finished products on site, often in sub-optimal storage conditions. The fact that the organism was observed to proliferate in the BMX system suggests that its presence, even at low levels, could be a potential concern in emulsified *Cannabis* oil formulations or edible products that are stored in closed containers.

Of greater potential concern than the bacterial growth is the failure of both culture-based TYM platforms to support efficient growth and detection of *Aspergillus* species, which were present in proportions of 18–58% of classified ITS2 reads at the genus level in 10/15 samples. Initially, it was suspected that the significant TYM qPCR and read counts might derive from dead cells, perhaps as a result of growers attempting to sterilize the plant material. Quantitative PCR data using active cultures grown in TSB, however, indicates that CFU counts from two *Aspergillus* species inoculated onto 3M TYM petri film were ~1000× lower than expected based on qPCR Cq values that accurately predict CFUs in other species ( Figure 3). Elevated Cq values due to ribosomal DNA copy number amplification does not seem a likely explanation because the estimated copy numbers of several *Aspergillus* species are similar to those of other fungi ^40, 41^. While the presence of spores with a slow germination rate ^42^ could explain the results on plant material, it does not explain the qPCR result using active cultures. Another factor could be the obligate hyphal growth nature of *Aspergillus* species ^43^, wherein each colony forming unit may contain hundreds of interconnected hyphal cells.

These findings are surprising, and therefore a third culture-based system, manufactured by Biolumix, was tested for its ability to detect *A. fumigatus* after 48 hours of growth at 26°C following inoculation from a saturated TSB culture. The result was negative. The failure of three different culture-based platforms to detect *Aspergillus* species suggests the need for caution in the use of such platforms. Validation data for the detection of *Aspergillus* on 3M rapid TYM Petri-film presented in 3M’s marketing material ^44^ is for culture at 25°C, whereas the instructions for use specify culture at room temperature (~4°C below 25°C). McClenny ^45^ recommends longer times and higher temperatures to accurately detect *Aspergilli* with culture based methods. The 3M films used in this study were incubated at 25 ± 1.0°C for 72 hours and still showed low efficacy detecting *Aspergilli*.

*Aspergillus* is arguably the most significant fungal threat in *Cannabis* cultivation. Aspergillosis has been reported in numerous immunocompromised patients and, to date accounts for the only clinical reports of fatalities associated with an infectious organism linked to *Cannabis* consumption ^16– 18, 46– 48^. Vonberg *et al.* demonstrated a 57% fatality rate for Aspergillosis in hospital-bound immunocompromised patients, while also demonstrating airborne infectability at or below 1 CFU/cubic meter ^49^. Growers may pasteurize *Cannabis* samples to avoid failing culture-based microbial testing, but *Aspergillus* spores are pasteurization resistant ^50^, as are the toxins they produce ^51^, so pasteurization does not eliminate the potential risk from these organisms.

Another interesting observation is the apparent growth inhibition of *Penicillium* species ( *P. citrinum, P. brevicompactum, P. olsonii* and *P. quercetorum*) in several samples with high proportions of *Trichoderma, Fusarium, Rhodotorula* or *Saccharomyces* reads after culture (samples 1,2,4,7 and 16). Other classified species that failed to grow in some of those samples include *Furcaspora eucalypti* and *Tilletiopsis pallescens*. Organic growth practices often utilize beneficial bacterial or fungal endophytes ^52^ to promote crop growth and to enable lower chemical fungicide use. For example, *Trichoderma* species are known to synthesize β-1,3 gluconases and a chitinase which work synergistically to break down the cell walls of other fungi ^53, 54^. The State of Nevada has issued guidelines for allowable pesticides for use in *Cannabis* cultivation that include various *Trichoderma* and *Bacillus* species ^55^. However, in most states, the use of such beneficial microbes may be precluded by the requirement for stringent yeast and mold testing that does not discriminate between beneficial and harmful microorganisms. More specific nucleic acid based testing techniques can resolve this. The FDA is moving in this direction for food safety testing with the GenomeTrakr Network ^56^.

Finally, as observed in a previous study on the *Cannabis* fungal microbiome in a different sample set ^15^, *P. citrinum* is highly prevalent in the samples tested here. This species has been isolated as a growth promoting endophyte in *Cannabis* and several other plant species ^10, 11, 57– 59^. *P. citrinum* produces the nephrotoxin citrinin, although it is not clear whether the presence of citrinin in *Cannabis* flowers or extracts represents an actual health threat. However, the high prevalence of *P. citrinum* in *Cannabis* samples suggests that it is an area worthy of further investigation.

These data have several limitations. Quantitative inter-sample comparisons cannot be performed with the sequencing data at present due to the lack of internal controls to help calibrate any pooling or sampling issues throughout the workflow. The qPCR data can be used to estimate inter-sample bacterial or fungal burden but these data do not always resolve to the genus or species level. Intra-sample comparisons can nonetheless provide information on the relative proportions of bacterial or fungal species. Sampling from BMX cards was straightforward, since it uses a liquid culture medium, but 3M sampling was subject to bias in scraping off colonies from culture plates. Additionally, the use of Nextera shearing and primer amplification may introduce some biases due to transposon integration preferences. The fragmentation approach is necessary to avoid ITS2 amplicon size bias in Illumina MiSeq clustering ^60, 61^.

## Conclusions

Culture based techniques used to measure the microbial burden and establish safety of *Cannabis* have several shortcomings. States adopt and implement regulations at different tolerance thresholds for bacteria and fungi without specifically detailing standardized methods or coordinating inter-laboratory ring testing. Yeast and mold counts from the culture-based platforms tested here are confounded by the growth of bacteria - even when antibiotics like chloramphenicol are included. The microbiome in the plant material tested changes radically after culturing, such that the microbes and counts that are finally observed bear little or no resemblance to those of the starting sample. This represents a classic observer effect, where the act of measuring the microbial composition using these culture-based methods fundamentally changes that composition - which is a well-studied phenomenon known as the “great plate-count anomaly” ^62^. This is a serious issue, which clearly has implications beyond Cannabis safety testing. The 3M and BMX platforms tested here are also used widely in the food testing industry.

Perhaps the most concerning observation is that one of the most regulated of fungal pathogens, *Aspergillus -* the only microbe to ever be associated with clinical harm concerning cannabis - grows poorly, and is therefore severely under-reported by current culture-based platforms. The differential growth of other toxigenic fungi, depending on the companion species present, further influences the results. Bacterial pathogens are not uncommon, and beneficial bacteria are also capable of influencing the growth or inhibition of other flora.

We have demonstrated that molecular testing is capable of accurately quantifying and identifying a wide spectrum of microorganisms present on *Cannabis* samples, while avoiding false positives due to the presence of bacteria for fungal testing. Molecular testing is rapid and is capable of distinguishing between harmful and beneficial microbes – permitting the use of the latter in organic cultivation practices to eliminate the need for reliance on chemical fungicides.

## Data availability

The data referenced by this article are under copyright with the following copyright statement: Copyright: © 2016 McKernan K et al.

Data associated with the article are available under the terms of the Creative Commons Zero "No rights reserved" data waiver (CC0 1.0 Public domain dedication). 

F1000Research: Dataset 1. Raw data of metagenomic analysis of medicinal Cannabis samples, 10.5256/f1000research.9662.d137123 ^63^

## References

[1] Kusari P Kusari S Lamshoft M: Quorum quenching is an antivirulence strategy employed by endophytic bacteria. *Appl Microbiol Biotechnol.* 2014;98(16):7173–83. 10.1007/s00253-014-5807-3 24846733

[2] Kusari P Kusari S Spiteller M: Implications of endophyte-plant crosstalk in light of quorum responses for plant biotechnology. *Appl Microbiol Biotechnol.* 2015;99(13):5383–90. 10.1007/s00253-015-6660-8 25971199

[3] Turner TR James EK Poole PS: The plant microbiome. *Genome Biol.* 2013;14(6):209. 10.1186/gb-2013-14-6-209 23805896 PMC3706808

[4] Compant S Clément C Sessitsch A: Plant growth-promoting bacteria in the rhizo- and endosphere of plants: Their role, colonization, mechanisms involved and prospects for utilization. *Soil Biol Biochem.* 2010;42(5):669–78. 10.1016/j.soilbio.2009.11.024

[5] Reinhold-Hurek B Hurek T: Living inside plants: bacterial endophytes. *Curr Opin Plant Biol.* 2011;14(4):435–43. 10.1016/j.pbi.2011.04.004 21536480

[6] Bonfante P Genre A: Mechanisms underlying beneficial plant-fungus interactions in mycorrhizal symbiosis. *Nat Commun.* 2010;1:48. 10.1038/ncomms1046 20975705

[7] Berendsen RL Pieterse CM Bakker PA: The rhizosphere microbiome and plant health. *Trends Plant Sci.* 2012;17(8):478–86. 10.1016/j.tplants.2012.04.001 22564542

[8] Winston ME Hampton-Marcell J Zarraonaindia I: Understanding cultivar-specificity and soil determinants of the *cannabis* microbiome. *PLoS One.* 2014;9(6):e99641. 10.1371/journal.pone.0099641 24932479 PMC4059704

[9] Houbraken J Frisvad JC Samson RA: Taxonomy of *Penicillium* section *Citrina*. *Stud Mycol.* 2011;70(1):53–138. 10.3114/sim.2011.70.02 22308046 PMC3233908

[10] Gautam A Kant M Thakur Y: Isolation of endophytic fungi from *Cannabis sativa* and study their antifungal potential. *Archives Of Phytopathology And Plant Protection.* 2013;46(6):627–35. 10.1080/03235408.2012.749696

[11] Kusari P Kusari S Spiteller M: Endophytic fungi harbored in *Cannabis sativa* L.: diversity and potential as biocontrol agents against host plant-specific phytopathogens. *Fungal Divers.* 2013;60(1):137–51. 10.1007/s13225-012-0216-3

[12] Chung EJ Hossain MT Khan A: *Bacillus oryzicola* sp. nov., an Endophytic Bacterium Isolated from the Roots of Rice with Antimicrobial, Plant Growth Promoting, and Systemic Resistance Inducing Activities in Rice. *Plant Pathol J.* 2015;31(2):152–64. 10.5423/PPJ.OA.12.2014.0136 26060434 PMC4453996

[13] Paz IC Santin RC Guimarães AM: Eucalyptus growth promotion by endophytic *Bacillus* spp. *Genet Mol Res.* 2012;11(4):3711–20. 10.4238/2012.August.17.9 22930432

[14] Shi Y Lou K Li C: Growth and photosynthetic efficiency promotion of sugar beet ( *Beta vulgaris* L.) by endophytic bacteria. *Photosynth Res.* 2010;105(1):5–13. 10.1007/s11120-010-9547-7 20405213

[15] McKernan K Spangler J Zhang L: *Cannabis* microbiome sequencing reveals several mycotoxic fungi native to dispensary grade *Cannabis* flowers [version 2; referees: 2 approved]. *F1000Res.* 2015;4:1422. 10.12688/f1000research.7507.2 27303623 PMC4897766

[16] Ruchlemer R Amit-Kohn M Raveh D: Inhaled medicinal cannabis and the immunocompromised patient. *Support Care Cancer.* 2015;23(3):819–22. 10.1007/s00520-014-2429-3 25216851

[17] Gargani Y Bishop P Denning DW: Too many mouldy joints - marijuana and chronic pulmonary aspergillosis. *Mediterr J Hematol Infect Dis.* 2011;3(1):e2011005. 10.4084/MJHID.2011.005 21625309 PMC3103256

[18] Bal A Agarwal AN Das A: Chronic necrotising pulmonary aspergillosis in a marijuana addict: a new cause of amyloidosis. *Pathology.* 2010;42(2):197–200. 10.3109/00313020903493997 20085530

[19] Taylor DN Wachsmuth IK Shangkuan YH: Salmonellosis associated with marijuana: a multistate outbreak traced by plasmid fingerprinting. *N Engl J Med.* 1982;306(21):1249–53. 10.1056/NEJM198205273062101 7070444

[20] Centers for Disease Control (CDC): Salmonellosis traced to marijuana--Ohio, Michigan. *MMWR Morb Mortal Wkly Rep.* 1981;30(7):77–9. 6789127

[21] Health DDoE: Special Concerns Associated with Marijuana Extractions, Concentrations, Infusions, and Infused Foods. Public Health Inspections Division.2016 Reference Source

[22] Marcu J: *Cannabis Inflorescence Cannabis* spp. Standards of Identity, Analysis, And Quality Control. American Herbal Pharmacopoeia.2013 Reference Source

[23] Schoch CL Seifert KA Huhndorf S: Nuclear ribosomal internal transcribed spacer (ITS) region as a universal DNA barcode marker for *Fungi*. *Proc Natl Acad Sci U S A.* 2012;109(16):6241–6. 10.1073/pnas.1117018109 22454494 PMC3341068

[24] Schoch CL Robbertse B Robert V: Finding needles in haystacks: linking scientific names, reference specimens and molecular data for Fungi. *Database (Oxford).* 2014;2014: pii: bau061. 10.1093/database/bau061 24980130 PMC4075928

[25] Ram JL Karim AS Sendler ED: Strategy for microbiome analysis using 16S rRNA gene sequence analysis on the Illumina sequencing platform. *Syst Biol Reprod Med.* 2011;57(3):162–70. 10.3109/19396368.2011.555598 21361774

[26] McKernan KJ Spangler J Helbert Y: DREAMing of a patent-free human genome for clinical sequencing. *Nat Biotechnol.* 2013;31(10):884–7. 10.1038/nbt.2703 24104751

[27] McKernan KJ Spangler J Zhang L: Expanded genetic codes in next generation sequencing enable decontamination and mitochondrial enrichment. *PLoS One.* 2014;9(5):e96492. 10.1371/journal.pone.0096492 24788618 PMC4008621

[28] Ounit R Lonardi S: Higher classification sensitivity of short metagenomic reads with CLARK-S. *Bioinformatics.* 2016; pii: btw542. 10.1093/bioinformatics/btw542 27540266

[29] Kõljalg U Nilsson RH Abarenkov K: Towards a unified paradigm for sequence-based identification of fungi. *Mol Ecol.* 2013;22(21):5271–7. 10.1111/mec.12481 24112409

[30] Edgar RC: UPARSE: highly accurate OTU sequences from microbial amplicon reads. *Nature methods.* 2013;10(10):996–8. 10.1038/nmeth.2604 23955772

[31] Edgar RC: Search and clustering orders of magnitude faster than BLAST. *Bioinformatics.* 2010;26(19):2460–1. 10.1093/bioinformatics/btq461 20709691

[32] McMurdie PJ Holmes S: Waste not, want not: why rarefying microbiome data is inadmissible. *PLoS Comput Biol.* 2014;10(4):e1003531. 10.1371/journal.pcbi.1003531 24699258 PMC3974642

[33] Hasan MM Rahman SM Kim GH: Antagonistic potentiality of Trichoderma harzianum towards seed-borne fungal pathogens of winter wheat cv. Protiva *in vitro* and *in vivo*. *J Microbiol Biotechnol.* 2012;22(5):585–91. 10.4014/jmb.1107.07063 22561850

[34] Abou-Zeid AM Altalhi AD Abd El-Fattah RI: Fungal control of pathogenic fungi isolated from wild plants in Taif Governorate, Saudia Arabia. *Roum Arch Microbiol Immunol.* 2007;66(3–4):90–6. 18928069

[35] Wurzer J: The Science of Cannabis. *CannMed.* 2016;2016 Reference Source

[36] Ounit R Wanamaker S Close TJ: CLARK: fast and accurate classification of metagenomic and genomic sequences using discriminative *k*-mers. *BMC Genomics.* 2015;16:236. 10.1186/s12864-015-1419-2 25879410 PMC4428112

[37] Kerepesi C Grolmusz V: Evaluating the Quantitative Capabilities of Metagenomic Analysis Software. *Curr Microbiol.* 2016;72(5):612–6. 10.1007/s00284-016-0991-2 26831696

[38] Hemashenpagam N Selvaraj T: Effect of arbuscular mycorrhizal (AM) fungus and plant growth promoting rhizomicroorganisms (PGPR's) on medicinal plant Solanum viarum seedlings. *J Environ Biol.* 2011;32(5):579–83. 22319872

[39] Zeiller M Rothballer M Iwobi AN: Systemic colonization of clover ( *Trifolium repens*) by *Clostridium botulinum* strain 2301. *Front Microbiol.* 2015;6:1207. 10.3389/fmicb.2015.01207 26583010 PMC4628109

[40] Herrera ML Vallor AC Gelfond JA: Strain-dependent variation in 18S ribosomal DNA Copy numbers in *Aspergillus fumigatus*. *J Clin Microbiol.* 2009;47(5):1325–32. 10.1128/JCM.02073-08 19261786 PMC2681831

[41] Black J Dean T Byfield G: Determining fungi rRNA copy number by PCR. *J Biomol Tech.* 2013;24(1):32–8. 10.7171/jbt.13-2401-004 23543828 PMC3523570

[42] Marín S Sanchis V Sáenz R: Ecological determinants for germination and growth of some *Aspergillus* and *Penicillium* spp. from maize grain. *J Appl Microbiol.* 1998;84(1):25–36. 10.1046/j.1365-2672.1997.00297.x 15244054

[43] Brand A: Hyphal growth in human fungal pathogens and its role in virulence. *Int J Microbiol.* 2012;2012:517529. 10.1155/2012/517529 22121367 PMC3216317

[44] Nordby T: Rapid Quantitative Enumeration of Yeasts and Molds.2013 Reference Source

[45] McClenny N: Laboratory detection and identification of *Aspergillus* species by microscopic observation and culture: the traditional approach. *Med Mycol.* 2005;43(Suppl 1):S125–8. 10.1080/13693780500052222 16110804

[46] Remington TL Fuller J Chiu I: Chronic necrotizing pulmonary aspergillosis in a patient with diabetes and marijuana use. *CMAJ.* 2015;187(17):1305–8. 10.1503/cmaj.141412 26100839 PMC4646751

[47] Chusid MJ Gelfand JA Nutter C: Letter: Pulmonary aspergillosis, inhalation of contaminated marijuana smoke, chronic granulomatous disease. *Ann Intern Med.* 1975;82(5):682–3. 10.7326/0003-4819-82-5-682 1094876

[48] Cescon DW Page AV Richardson S: Invasive pulmonary aspergillosis associated with marijuana use in a man with colorectal cancer. *J Clin Oncol.* 2008;26(13):2214–5. 10.1200/JCO.2007.15.2777 18445848

[49] Vonberg RP Gastmeier P: Nosocomial aspergillosis in outbreak settings. *J Hosp Infect.* 2006;63(3):246–54. 10.1016/j.jhin.2006.02.014 16713019

[50] Fujikawa H Itoh T: Tailing of thermal inactivation curve of *Aspergillus niger* spores. *Appl Environ Microbiol.* 1996;62(10):3745–9. 8837430 10.1128/aem.62.10.3745-3749.1996 PMC168182

[51] Carvajal M Bolaños A Rojo F: Aflatoxin M1 in pasteurized and ultrapasteurized milk with different fat content in Mexico. *J Food Prot.* 2003;66(10):1885–92. 14572228 10.4315/0362-028x-66.10.1885

[52] Afzal R Shinwari ZK Iqrar I: Selective Isolation and Characterization of Agriculturally Beneficial Endophytic Bacteria from Wild Hemp using Canola. *Pak J Bot.* 2015;47(5):1999–2008. Reference Source

[53] Ulhoa CJ Peberdy JF: Regulation of chitinase synthesis in *Trichoderma harzianum*. *J Gen Microbiol.* 1991;137(9):2163–9. 10.1099/00221287-137-9-2163 1748872

[54] Harman GE: Overview of Mechanisms and Uses of *Trichoderma* spp. *Phytopathology.* 2006;96(2):190–4. 10.1094/PHYTO-96-0190 18943924

[55] Barbee J: Medical Marijuana Pesticide List (Revised 05/02/2016). *State of Nevada Department of Agriculture.* 2016 Reference Source

[56] FDA: Genome Trakr Network. Reference Source

[57] Vega FE Posada F Peterson SW: *Penicillium* species endophytic in coffee plants and ochratoxin A production. *Mycologia.* 2006;98(1):31–42. 10.3852/mycologia.98.1.31 16800302

[58] Khan AL Al-Harrasi A Al-Rawahi A: Endophytic Fungi from Frankincense Tree Improves Host Growth and Produces Extracellular Enzymes and Indole Acetic Acid. *PLoS One.* 2016;11(6):e0158207. 10.1371/journal.pone.0158207 27359330 PMC4928835

[59] Khan SA Hamayun M Yoon H: Plant growth promotion and *Penicillium citrinum*. *BMC Microbiol.* 2008;8:231. 10.1186/1471-2180-8-231 19099608 PMC2631606

[60] Fadrosh DW Ma B Gajer P: An improved dual-indexing approach for multiplexed 16S rRNA gene sequencing on the Illumina MiSeq platform. *Microbiome.* 2014;2(1):6. 10.1186/2049-2618-2-6 24558975 PMC3940169

[61] Pinto AJ Raskin L: PCR biases distort bacterial and archaeal community structure in pyrosequencing datasets. *PLoS One.* 2012;7(8):e43093. 10.1371/journal.pone.0043093 22905208 PMC3419673

[62] Hugenholtz P: Exploring prokaryotic diversity in the genomic era. *Genome Biol.* 2002;3(2):REVIEWS0003. 10.1186/gb-2002-3-2-reviews0003 11864374 PMC139013

[63] McKernan K Spangler J Helbert Y: Dataset 1 in: Metagenomic Analysis of Medicinal *Cannabis.* Samples; Pathogenic Bacteria, Toxigenic Fungi, and Beneficial Microbes Grow in Culture-Based Yeast and Mold Tests. *F1000Research.* 2016 Data Source 10.12688/f1000research.9662.1 PMC5089129 27853518

