# Supplementary material for: Metagenomic analysis of medicinal Cannabis samples; pathogenic bacteria, toxigenic fungi, and beneficial microbes grow in culture-based yeast and mold tests
Source: F1000Res. 2016 Oct 7;5:2471. [Version 1] doi: 10.12688/f1000research.9662.1 (PMC5089129; doi:10.12688/f1000research.9662.1)
Supplement: Peer review history

# Peer review: Referee response for version 1

**DOI:** 10.5256/f1000research.10411.r16858

**Published:** 2016-11-07

**Authors:** Jahan Marcu

**Affiliations:** 1 Americans for Safe Access (ASA), Washington, DC, USA

This article represents an area of research that needs more attention. My only concerns are minor, and are regarding the figures in the article. The figures do not have any error bars/indication of replicability. It would be great if there were more reproducibility indicated within the figures, as this article will be highly read and potentially utilized in a growing industry.

I have read this submission. I believe that I have an appropriate level of expertise to confirm that it is of an acceptable scientific standard.

# Peer review: Referee response for version 1

**DOI:** 10.5256/f1000research.10411.r16859

**Published:** 2016-10-18

**Authors:** Philippe Henry, Lukas Wille

**Affiliations:** 1 Ecosystem Science and Management program, College of Science and Management (CSAM), University of Northern British Columbia (UNBC), Prince George, BC, Canada; 2 Canada & Leysin Scientific, Kelowna, BC, Canada; 3 Department of Crop Sciences, Research Institute for Organic Agriculture, Frick, Switzerland

We would like to extend our sincere gratitude for the opportunity to provide an open peer review for the work of McKernan and colleagues on the Cannabis microbiome and uses of metagenomics to shed light on the microbial complement of the Cannabis phyllosphere. As strong proponents of open science, we engage to provide an objective assessment of the work presented here and to make suggestions aimed at improving the clarity and readability of the present work.

 The microbiome (the collection of microbial genomes present on an organism or in an environment) has emerged as an additional dimension (in addition to genomic, epigenomic, metabolomic and phenotypic data…) from which one can harness cryptic information that may contribute to a particular biological phenomenon. Novel technical advances have enabled a metagenomic approach in which one can isolate and enumerate bacterial and fungal genus/species from environmental samples. In their paper, McKernan *et al. *compare the performance of traditional culture based techniques with commercial qPCR kits in terms of accuracy and ability to detect different types of microbes, with an emphasis on prokaryotic organisms, which was so far underrepresented in previous published work (including a recent F1000 research paper by the same lead author).

 We support the authors’ work and understand that as pioneers in the Cannabis microbiome space, they are currently laying the foundation on which further development of assays will likely rely strongly. As such, much of our comments relate to improving the transparency of their results. We pose several questions/comments, which the author may choose to incorporate into updated versions of this paper. Overall, the paper is very well written and we do not have any editorial suggestions, except for the spelling of Biomerieux, which in one instance requires the accent aigu: “é”. Below, please find minor comments, which we would like to authors to consider:

- We are in agreement with review 1 (Ethan Russo) that the abstract could be improved if word limit permits. We particularly think that the concluding statement could incorporate a stronger statement about the application of their approach in the Cannabis industry.
- A short statement on why the comparison between culture-based platforms and DNA-based detection is relevant (e.g. in “Background”) would be of general interest to the readership.

 

Introduction

- We found the introduction to lack a common thread and rather abruptly “jumps” between the primary objectives of the paper: methodological comparison between different microorganism detection techniques, microbe-microbe interactions and health implications: e.g. in paragraph 1 the authors focus on microbiome-ecological considerations. Then again in paragraph 3, they bring up plant-microbe/microbe-microbe interaction although the rest of this paragraph is focus on methodologies.
- The statement *”…no studies have examined the impact of beneficials…”* does not make much sense there. Perhaps the considerations about *Trichoderma* could be saved for the discussion.
- Some brief background on the two culture platforms (3M, BMX) would help frame the need for other novel technologies in microbial detection.  

Methods

- As a general comment to all sections from hereon, it would be helpful to have the same sub-headings (as much as possible) logically flow from methods to results and into the discussion points:

e.g.  

1. DNA-extraction from plants
2. Platform culturing
3. qPCR
4. Metagenomic / sequencing

 

 (Currently, the last paragraph of the Introduction gives an overview of the methods, the first technique that is mentioned is NGS, then qPCR; in the Methods and Results it is first qPCR and then NGS…)

 The above-mentioned will help disentangle some of the concepts introduced here, and send a clearer message to the readership: why and when to use qPCR/metagenomics instead of conventional approaches.

 If there is sufficient evidence that the novel approach outperforms the old is another question that seems rather elusive in the current paper.

- It would be valuable to share some information about their standard curve and how they derived their Cq values of 21 and 26 cycles for TYM and TAC assays respectively. This brings up the point that a more convincing comparison of culture based and qPCR assays need to be provided in order for the readership to assess if/when one should be used over another.
- It would be useful to share the name of the R package used to undertake the USEARCH OTUs analyses.
- The authors should consider perhaps using other multivariate statistics than bivariate correlation coefficients. Another angle to tackle this is to implement diversity analyses on the microbial community data using either Shannon’s diversity index or some other metric such as alpha- and beta- diversity in each sample. While sample size is likely limiting, are there other similarities between samples for common origins? Or grown in similar conditions?
- Please expand on what you mean by “specificity analysis” and how this was undertaken.

 

Results

- The title of the first section is misleading: “qPCR and colony counts before and after”; i.e. there is no colony count before (refer to above comment about unifying sub-heading in paper sections).
- Table 1 could be presented in a clearer way: Table description and content (the column headers) are redundant; too much text in the table description; instead of sample nr., give some info in the sample identification (e.g. origin); visually separate TYM and TAC.
- The presentation of the results (using excel bar plots), while understandable, is not that efficient at presenting the data at hand. Without overstepping, we suggest looking at multivariate plots that would be more suited to drive their points home.

 

Discussion

- It seems counterintuitive that qPCR, being more sensitive than plating approach, would fail the lowest number of samples out of all approaches: Is the BMX positive bias toward *C. botulinum* a false positive or is the lack of *C. botulinum* detection by qPCR a false negative?
- In that vein, it would be helpful to describe your strategy to assess false positives, i.e. how many negative controls were implemented? Any negative control with botulism?
- Failure thresholds are subjective in nature, please expand on how the Cq threshold is superior, what microbial load (e.g. ng of DNA) do each of the two Cq thresholds correspond to?
- We found that while a large and varied bacterial assemblage was identified here, it would be important to note that modern Cannabis such as the 15 samples presented here have likely gone through several genetic and microbiotic bottlenecks. A recent paper on the Agave microbiome ^1^ demonstrates the paucity of the microbiome in domesticated/farmed plants compared to wild relatives, likely due to the pervasive effects of monoculture. While the Cannabis domestication process is convoluted and masked by prohibition, it is likely that the same pattern is observed in Cannabis. Characterizing the genetic profiles of Cannabis, along with the microbiome of wild Cannabis accessions will likely yield enhanced inference in terms of the underlying mechanisms related to plant growth and disease tolerance.
- A larger part of the discussion should be dedicated to the community composition shift before and after culturing. Especially some considerations about the biological relevance of this shift: i.e. are the pathogens that grow on the plate relevant for human health/plant growth considerations? Or the other way round: are the pathogens detected before culturing relevant for human health/plant growth issues? It would be good to more explicitly separate what the authors think are artifacts caused by different methodologies (community shifts) with biologically relevant phenomena.
- If the authors found polymorphisms in OTUs, they may want to suggest the application of the Cannabis microbiome to provide higher resolution to clustering exercises in highly related or poly-hybridized Cannabis accessions. This may also be used to trace the origin of particular dispensary samples to a cultivator or methodology of plant growth as using hydroponics, soil, aquaponics, etc. will likely influence the Cannabis rhizospheres and phyllospheres. 
- While the authors discuss the presence of *C. botulinum* and *Aspergillum* spp. in emulsified oils, perhaps addressing the use of extraction technologies such as butane, propane, Nitrogen, CO _2_, Rosin tech and its putative impact on the presence of unwanted microorganisms may be a topic of general interest. In Canada until recently, only Cannabis flowers were prescribed as medicinal Cannabis. This study highlights some drawbacks of using this type of Cannabis for medical purposes, particularly when used in immune-compromised individuals, and indirectly supports the use of Cannabis extracts that can be dosed effectively with minimal risks of exposure to toxicogenic microbes.

 

 We sincerely hope that the authors will find our review useful and we remain available for further discussion through the F1000 research platform.

 Kind regards,

 Philippe Henry and Lukas Wille

We have read this submission. We believe that we have an appropriate level of expertise to confirm that it is of an acceptable scientific standard.

## References

[1] Coleman-DerrDDesgarennesDFonseca-GarciaCGrossSClingenpeelSWoykeTNorthGViselAPartida-MartinezLPTringeSG: Plant compartment and biogeography affect microbiome composition in cultivated and native Agave species. *New Phytol*.2016;209(2) : 10.1111/nph.13697 798-811 10.1111/nph.13697 26467257PMC5057366

# Peer review: Referee response for version 1

**DOI:** 10.5256/f1000research.10411.r16857

**Published:** 2016-10-17

**Authors:** Justin Fischedick

**Affiliations:** 1 Excelsior Analytical Lab, Inc., Union City, CA, USA

The purpose of this study was to investigate the composition of microorganisms found on cannabis samples and compare the ability of different culture based testing platforms with a qPCR method. Although the study provides some valuable data into some of the short comings of culture based methods it has some experimental design weaknesses that make it difficult to draw strong conclusions from this data set. 

 Introduction:

 Since the purpose of the study is to discuss the difference between a qPCR based microbial testing platform with culture based methods the introduction should focus more on discussing this in other industries. For example it's becoming well known that only a small percentage of organisms that exist in nature are easily cultured on the most common forms of media used. Rapid advances in sequencing are allowing metagenomic analysis of soil and plant microbiomes which also demonstrates the limitations of culturing methods. Issues like specificity between qPCR and culture based methods should be highlighted. 

 Methods: 

 Plant material - Nowhere in the methods section is any information provided about the cannabis plant material. Was it cannabis flowers? Were they dried? Was the sample homogenized in anyway? 

 Information about all the samples used in this study should be summarized in a table or in a section within the methods part of the manuscript. 

 Results: 

- Table 1- This table highlights one of the main criticisms I have with this study. There are no replicates. These results seem to be based off a single analysis of each sample. Therefore we can't conclude anything about the reproducibility of the qPCR platform compared to the other platforms.
- It is also interesting to note that most of the culture based methods detected levels of fungi that would be considered failures while most of the qPCR samples detected only low levels of fungal DNA. 
- It is difficult to follow from reading through the text of the manuscript which samples were analyzed by metagenomic sequencing. Every sample analyzed by metagenomic sequencing and a summary of their results, in terms of what species were detected and their approximate amounts, should be summarized somewhere in the manuscript for ease of reference and completeness of data presentation. Why those samples were chosen should be discussed. 
- Throughout the results section numbers of samples are discussed but we don't know if those are the same samples or which samples shown in Table 1.
  For example page 7 paragraph 2:
  "First, we observed that although *Aspergillus* species were present in 15 plant samples (average proportion: 25% of classified reads), they were only detected at low levels in three samples after culturing on either 3M or BMX media (average proportion: 1.1% or 0.4% of classified reads, respectively).".
  Which 15 plant samples? This kind of vague reference to samples needs to be corrected and be made more clear. 
  Page 6, sequence reproducibility:
  "14 samples".
  Which samples? 
  Page 6, paragraph 4:
  "Pairs of samples from three of the seven growers....".
  Which detected fungi were commonly found and correlated? 
  Page 7, Figure 1:
  Why just discuss 2 and 14? Why not 7-13 or 15-16? 

To summarize I would like to see more error bars (or +-) from replicates and more complete summaries of data and samples information. 

I have read this submission. I believe that I have an appropriate level of expertise to confirm that it is of an acceptable scientific standard, however I have significant reservations, as outlined above.

# Peer review: Referee response for version 1

**DOI:** 10.5256/f1000research.10411.r16862

**Published:** 2016-10-10

**Authors:** Ethan Russo

**Affiliations:** 1 PHYTECS, Los Angeles, CA, USA

This is a very interesting, well written and designed account comparing the accuracy and utility of genetic microbial testing as compared to standard microbiological culture techniques. All aspects of study design, methods and conclusions are well explained and defended, and should easily allow replication if comparable techniques are applied.

 I would suggest expansion of the study's implications in the abstract if the word count will permit this.

 In 2005, Vancouver Coastal Health in British Columbia reported transmission of meningococcal cases by sharing of joints, and perhaps this pathogen deserves scrutiny given its ubiquity in young adults very likely to be engaging in social cannabis usage.

 The legal analysis permitting cross-border transmission of DNA from cannabis material has important implications for greater adoption of similar analytical techniques, which certainly seems warranted given the advantages in accuracy in distinguishing beneficial, commensal and symbiotic microbiota from pathogens, and the speed of this approach to the issue.

I have read this submission. I believe that I have an appropriate level of expertise to confirm that it is of an acceptable scientific standard.
